# Interaction between Saikosaponin D, Paeoniflorin, and Human Serum Albumin

**DOI:** 10.3390/molecules23020249

**Published:** 2018-01-27

**Authors:** Guo-Wu Liang, Yi-Cun Chen, Yi Wang, Hong-Mei Wang, Xiang-Yu Pan, Pei-Hong Chen, Qing-Xia Niu

**Affiliations:** 1 Department of Pathophysiology, Key Immunopharmacology Laboratory, Institute of Inflammation and Immune Diseases, Shantou University Medical College, Shantou 515000, China; gwliangizzy@163.com (G.-W.L.); posywy@163.com (Y.W.); junebulu@163.com (H.-M.W.); fxmdp9@163.com (X.-Y.P.); 2 Department of Pharmacology, Traditional Chinese Medicine Laboratory, Shantou University Medical College, Shantou 515000, China; chenyicun@yeah.net (Y.-C.C.); ph553253347@163.com (P.-H.C.)

**Keywords:** saikosaponin, paeoniflorin, human serum albumin, spectroscopy, molecular docking

## Abstract

Saikosaponin D (SSD) and paeoniflorin (PF) are the major active constituents of *Bupleuri Radix* and *Paeonia lactiflora Pall*, respectively, and have been widely used in China to treat liver and other diseases for many centuries. We explored the binding of SSD/PF to human serum albumin (HSA) by using fluorospectrophotometry, circular dichroism (CD) and molecular docking. Both SSD and PF produced a conformational change in HSA. Fluorescence quenching was accompanied by a blue shift in the fluorescence spectra. Co-binding of PF and SSD also induced quenching and a conformational change in HSA. The Stern-Volmer equation showed that quenching was dominated by static quenching. The binding constant for ternary interaction was below that for binary interaction. Site-competitive experiments demonstrated that SSD/PF bound to site I (subdomain IIA) and site II (subdomain IIIA) in HSA. Analysis of thermodynamic parameters indicated that hydrogen bonding and van der Waals forces were mostly responsible for the binary association. Also, there was energy transfer upon binary interaction. Molecular docking supported the experimental findings in conformation, binding sites and binding forces.

## 1. Introduction

Human serum albumin (HSA), a non-glycosylated single polypeptide chain (585 amino acid residues, 66500 Da) with α-helical secondary and a heart-shaped tertiary structure, accounts for about 60% of the total plasma protein, with a concentration of 0.05 g/mL in the bloodstream [1,2]. As a carrier, the protein acts to combine, transport and distribute a variety of endogenous and exogenous substances, such as metabolites, neurotransmitters, amino acids, hormones and drugs [3]. *Radix Bupleuri* (Chai Hu in Chinese) is derived from the dried roots of *Bupleurum chinense* DC. and *Bupleurum scorzonerifolium* Willd. Chai Hu contains many pharmacological ingredients, such as volatile oils, triterpenoid saponins, and flavonoids [4,5]. Among the triterpenoid saponins, which are the major active components of Chai Hu, the action of saikosaponin D (SSD, CAS Registry Number: 20874-52-6, Figure 1) is considered to be strongest. Following oral administration of *Bupleurum* dropping pills, C_max_, T_max_ and t_1/2_ of SSD in plasma of rats has been determined to be 3.93 ± 2.74 ng/mL, 0.33 ± 0.20 h, and 3.27 ± 1.78 h, respectively [6]. Recent studies report that SSD possesses anti-oxidative, anti-inflammatory and anti-fibrotic properties, and promotes apoptosis of hepatic stellate cells [7,8,9,10,11]. *Paeonia lactiflora Pall* (Shao Yao in Chinese) has widely been used in China for many centuries to treat many diseases, such as pyretic, allergic, and inflammatory disorders. Paeoniflorin (PF, CAS Registry Number: 23180-57-6, Figure 1), a water-soluble monoterpene glycoside, is purified from the dried root of Shao Yao. After oral administration of PF to rats, the C_max_, T_max_ and t_1/2_ of PF in plasma has been reported to be 809.16 ± 163.75 ng/mL, 14.65 ± 1.20 min, and 174.60 ± 94.52 min [12]. As a major compound of Shao Yao, PF efficiently alleviates oxidative damage in HUVECs via the ROS-HIF-1α/VEGF pathway, liver fibrosis by the HIF-1α/mTOR signaling pathway, non-alcoholic fatty liver disease through multiple signaling pathways, and inflammatory response in ulcerative colitis via inhibiting the MAPK/NF-κB pathway and apoptosis in mice [13,14,15,16].

The pharmacological activity of drugs is influenced by how they are interact with HSA and other target molecules in different tissues [17,18]. Little information on the co-binding of PF and SSD to HSA is available, although Chai Hu and Bai Shao have together been used for centuries in China and other Asian countries for liver diseases, gynecological diseases, and other diseases. Thus, the investigation of the binding between SSD/PF and HSA is of important significance in life sciences, chemistry, and medicine [19,20,21,22,23]. A previous study reported that PF alone at high concentrations binds HSA [24]. The present study is focused on the binding of SSD and PF at low concentrations with HSA. By comparison, there is a considerable difference in spectra, binding sites, and thermodynamic parameters of HSA induced by PF at low and high concentrations.

## 2. Results and Discussion

### 2.1. SSD- and PF-Induced Conformational Changes in HSA

#### 2.1.1. Fluorescence Spectra

**Figure 2:**
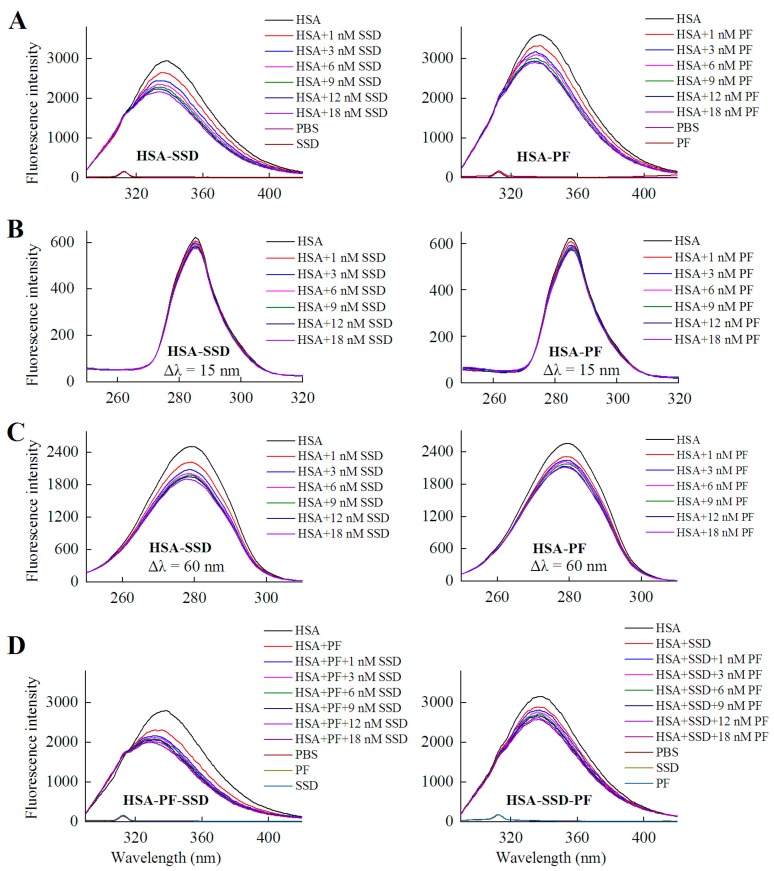
Fluorescence spectra of HSA in the presence of SSD and PF. Both conventional (**A**, **D**) and synchronous fluorescence measurements (**B**, **C**) were performed at 30 °C. C_HSA_ = 1 μM; in the ternary binding studies: C_PF_ = 3 μM; C_SSD_ = 3 μM; HSA, human serum albumin; PF, paeoniflorin; SSD, saikosaponin D; Δλ = 15 nm or Δλ = 60 nm for tyrosine and tryptophan residues, respectively.

HSA itself has endogenous fluorescence, primarily resulting from tyrosine and tryptophan residues. According to a previous report and our experiments, an excitation wavelength of 280 nm was employed [25,26]. As shown in Figure 2 and Table 1, examination of fluorescence peaks revealed a 5 nm blue shift for HSA following binding to SSD, and a 3 nm blue shift for HSA following PF binding. Unlike to these findings, a blue shift for HSA-PF at high-dose binding was not reported [24]. Although the SSD-induced shift was slightly greater to that of the PF-induced shift, this suggests that SSD and PF both induce conformational changes in HSA. Further, synchronous fluorescence measurements were performed to investigate that the two residues play a role in the fluorescence of HSA. A Δλ = 15 nm or Δλ = 60 nm is characteristic of the fluorescence spectra for tyrosine or tryptophan residues, respectively. A blue micro-shift of 2 nm in fluorescence peaks was observed at Δλ = 60 nm in HSA-SSD and HSA-PF binding, whereas there was no Δλ = 15 shift of peaks in the two binary binding. We then examined the interaction among the three components in a ternary reaction. As for HSA-PF-SSD binding, following the addition of PF at a fixed dose, titration with SSD induced a blue shift of 13 nm in the fluorescence peaks of HSA. As for HSA-SSD-PF binding, following the addition of SSD at a fixed dose, titration with PF only induced a slight blue shift of fluorescence spectra. It is interesting that the shift induced by HSA-PF-SSD binding was significantly above that induced by HSA-SSD plus HSA-PF binding, whereas the shift induced by HSA-SSD-PF binding was smaller than that induced by HSA-SSD plus HSA-PF binding. This indicates that SSD can greatly promote PF-induced conformational change of HSA, whereas PF has little effect on SSD-induced conformational change. This could be due to differences in binding affinity between HSA-SSD and HSA-PF. In short, the results indicate that the interaction between HSA and SSD/PF perturbs the microenvironment surrounding tryptophan. 

The shifts in the fluorescence peaks of HSA are closely related to conformational changes of the protein. A blue shift indicates that the hydrophobicity of amino acid residues increases in the microenvironment surrounding the fluorophores, and the structure of the HSA hydrophobic cavity becomes closer [27]. Therefore, the conformation of HSA is changed by binding to SSD and PF, individually and combined, and tryptophan residues are likely involved in the binding reaction [28].

**Figure 3:**
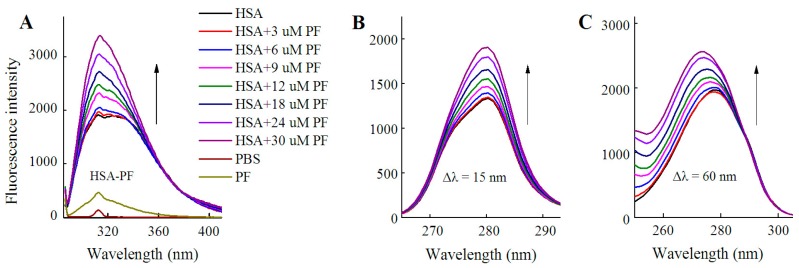
Fluorescence spectra of HSA in the presence of PF at 3–30 μM. Both conventional (**A**) and synchronous fluorescence measurements (**B**, **C**) were performed at 20 °C. C_HSA_ = 1 μM; C_PF_ = 0 (black), 3 (red); 6 (blue), 9 (pink), 12 (green), 18 (dark blue), 24 (purple), and 30 μM (brown), respectively.

As shown in Figure 3, fluorescence spectra of HSA were recorded for PF at 0–30 μM. It is worth noting that the fluorescence intensity did not decline, but rather significantly increased with the addition of PF. The peak appearance of HSA shifted to blue wavelengths although a blue shift at maximum emission was not observed. At Δλ = 60 nm, a blue shift of 5 nm (from 278 to 273 nm) formed at maximum emission. Undoubtedly, the difference between the present and reported study is mainly attributed to the concentration of PF [24]. These findings reveal that the amino acid residues in the vicinity of the fluorophore are disturbed by PF at both low and high concentrations. Therefore, PF at low concentrations ranging from 1 to 18 nM was employed in further study.

**Figure 4:**
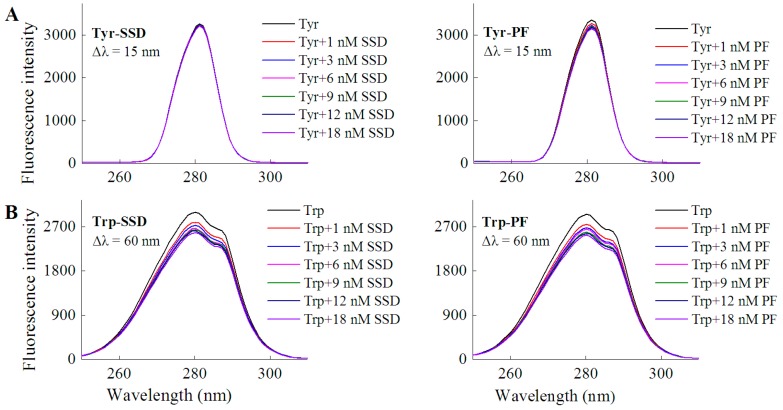
Fluorescence spectra of L-tyrosine (**A**) or L-tryptophan (**B**) in the presence of SSD/PF. Tyr, L-tyrosine ; Trp, L-tryptophan; C_Tyr_ = 10 μM; C_Trp_= 10 μM.

In order to investigate if a direct interaction is involved between the two drugs and tyrosine/tryptophan, the corresponding fluorescence measurements were carried out. In line with the quenching effect of both SSD and PF on HSA at Δλ = 15 nm and Δλ = 60 nm (Table 1), the results of the direct interaction support that tryptophan mainly contributed to the binding reaction (Table 1, Figure 4). Similarly, paeonol, a phenolic compound also derived from the paeony, was found to have direct competition with l-tryptophan [29].

#### 2.1.2. Circular Dichroism Studies

Circular dichroism (CD) was employed in order to confirm whether SSD or PF evokes a conformational change in HSA. HSA showed a negative absorption at 209 and 220 nm, consistent with a previous report [30]. The results can be calculated with the following equations [30]:

(1) $$MRE=\frac{CD\left(m\deg\right)}{10C_{p}nl}$$

(2) $$\alpha -helix(\%)=\frac{-{\left(MRE\right)}_{209}-4000CD\left(m\deg\right)}{33000-4000}\times 100$$

Here, *MRE*, *C_p_*, *n* and *l* represent the mean residue ellipticity, the concentration of HSA, the number of amino acid residues and the path length, respectively. In Equation (2), 33,000 and 4000 represent the *MRE* values of a pure α-helix and a β-form with random coil conformation at 209 nm, respectively.

**Figure 5:**
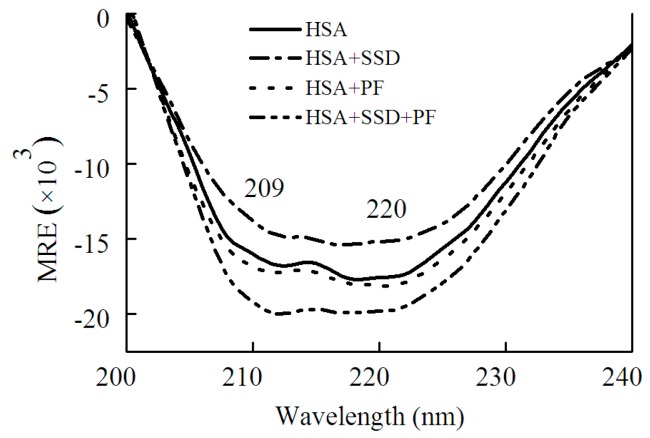
CD spectra of HSA in the absence and presence of SSD or PF.

As shown in Figure 5 and Table 2, SSD perturbed α-helices and β-turns, while it had little effect on β-sheets and random coils. In PF-HSA binding, PF at 1 μM slightly increased the α-helical content. On the contrary, PF at 25 μM slightly decreased the α-helical content, as previously shown [24]. In short, PF alone has a little effect on the helical structure of HSA. However, for ternary binding, the α-helical content increased significantly. Obvious differences were found between the binary and ternary complexes, suggesting that there is an additive effect of SSD and PF. These results are in agreement with those obtained from the investigations on HSA fluorescence spectra, further illustrating that HSA conformation is altered by SSD and PF.

### 2.2. Fluorescence Quenching of HSA by SSD and PF

As for HSA fluorescence intensity, our findings are consistent with prior studies showing the fluorescence of 1.5 μM HSA is about 4000 [31]. However, the fluorescence of 1 μM HSA in the present study was much above that (<1200) of 10 μM HSA [24]. This significant difference may mainly be ascribed to HSA itself, set-up of parameter measurements, sensitivity of light, filter, or detector, reaction system, and experimental conditions. As shown in Figure 2 and Table 1, the fluorescence intensity of HSA decreased with the increase in SSD or PF concentration. The percentage of synchronous fluorescence quenching at Δλ = 60 nm was clearly above that at Δλ = 15 nm. The direct interaction between the two drugs and tryptophan showed that the quenching effect at Δλ = 60 nm was stronger than that at Δλ = 15 nm (Figure 4 and Table 1), supporting that tryptophan is mainly responsible for the fluorescence quenching of HSA. In contrast, the quenching of HSA at Δλ = 60 nm induced by PF was a bit lower than that induced by SSD, which may suggest that HSA-SSD binding can exert a slightly stronger effect on tryptophan than HSA-PF binding.

### 2.3. Static Quenching of HSA Induced by SSD and PF

Fluorescence quenching takes place by two mechanisms, dynamic quenching and static quenching. Dynamic quenching is unaffected by the structure and bioactivity of the protein, and depends on intermolecular collision between the quencher and the fluorescent molecule at an excited state. Static quenching, which forms a new composite, is due to the intramolecular interaction of quenchers with fluorescent molecules at a ground state [32]. In order to probe the mechanism of SSD- and PF-mediated quenching, the Stern-Volmer equation was employed [25]:

(3) $$F_{0}/F=1+K_{q}\tau_{0}\left[Q\right]=1+K_{SV}\left[Q\right]$$

Here, *F*_0_ and *F* denote the fluorescence intensities in the absence and presence of SSD or PF, respectively. [*Q*] is for the concentration of SSD or PF. *K*_sv_, the Stern-Volmer quenching constant, reflects the degree of quencher contact with fluorescent molecules and reaction speed. The value of τ_0_, the average lifetime of fluorescent molecules, in the absence of quencher, is generally about 10^−8^ s. *K*_q_, the apparent bimolecular quenching rate constant. For the biomacromolecule, the maximum *K*_q_ value of most quenchers is 2.0 × 10^10^ L·mol^−1^·s^−1^ [25].

**Figure 6:**
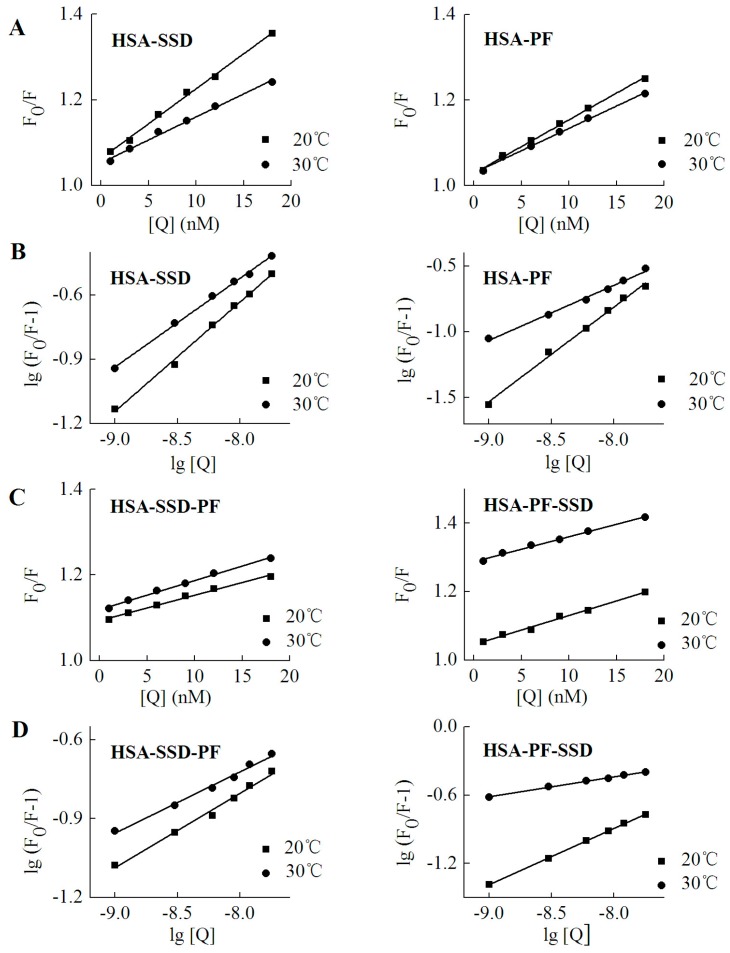
Stern-Volmer plots for HSA-SSD and -PF interaction. Experiments were performed in Figure 2. (**A**,**C**) are for Stern-Volmer plots. (**B**,**D**) represent double logarithmic plots for lg (F_0_/F-1) *vs*. lg [Q]. F_0_, HSA fluorescence intensities without the quencher; F, HSA fluorescence intensities with the quencher. [Q] denotes SSD or PF at doses of 0, 1, 3, 6, 9, 12 and 18 nM, respectively.

As shown in Table 3, the *K*_q_ at both 20 °C and 30 °C was much above 2.0 × 10^10^ L·mol^−1^·s^−1^, which indicates that SSD and PF induce static quenching. In ternary interaction, similar results occurred. Accordingly, Stern-Volmer plots for both binary and ternary interaction show that there is a good linear relationship between the concentration of drugs and the value of *F*_0_/*F*, confirming that static quenching of HSA can be induced by SSD or PF (Figure 6). In PF-HSA binding, it is interesting that the *K*_q_ induced by PF at 1–18 nM (~1 × 10^15^) was much above that induced by PF at 2.5–12.5 μM (~1 × 10^12^) [24]. This is most likely due the fact that PF at high concentrations produces more complex effects because more amino acid residues are perturbed.

### 2.4. Binding Constants, Sites and Forces

#### 2.4.1. Binding Constants and Numbers of Binding Sites

For static quenching, the binding constant and number of binding sites can be calculated by Equation (4) [25]:

(4) $$\mathrm{lg}\left(F_{0}/F-1\right)=\mathrm{lg}K_{a}+n\mathrm{lg}\left[Q\right]$$

The binding constant (*K_a_*) is a characteristic parameter that reflects the fastness between drug and the protein. The *K_a_* for low affinity binding is lower than 10^3^ mol·L^−1^ [26,32]. As shown in Table 4, there was a weak interaction between both HSA and SSD/PF. The resulting *K_a_* value and the number of binding sites (*n*) at 30 °C were below that at 20 °C, which is perhaps due to higher temperatures resulting in molecular diffusion and the dissociation of weakly bound complexes. The *K_a_* declined with increasing temperature, indicating that temperature is a major factor influencing the *K_a_*. A *K_a_* for low affinity binding is helpful for releasing the drug from the conjugates in target tissues, which affects the free fraction of the drugs to promote the drug effects [33]. Similar to our present study, HSA binding to PF or paeonol in previous studies also show a *K_a_* of 10^3^~10^4^ (Table 5). SSC is also, a triterpenoid saponin derived from Chai Hu. The *K_a_* of SSC with HSA is also on the order of 10^3^, in line with SSD (Table 5). In ternary binding, the *K_a_* and the number of binding sites in the ternary interaction were below those in binary binding, suggesting that release of the drugs is easier upon ternary interaction than upon binary interaction. 

#### 2.4.2. Identification of SSD or PF Binding Sites

**Figure 7:**
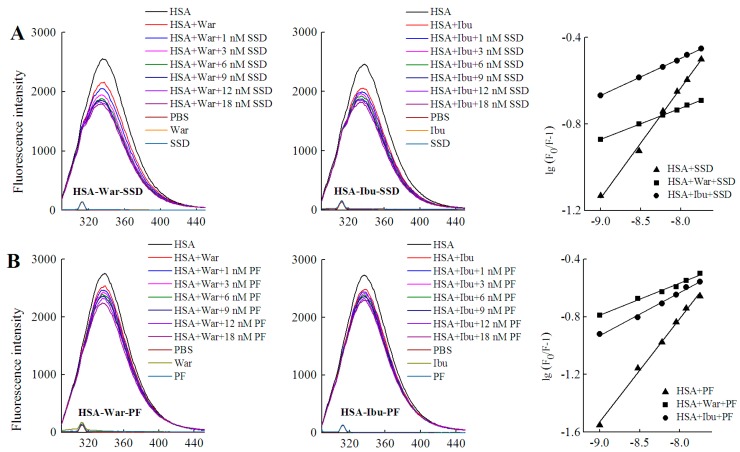

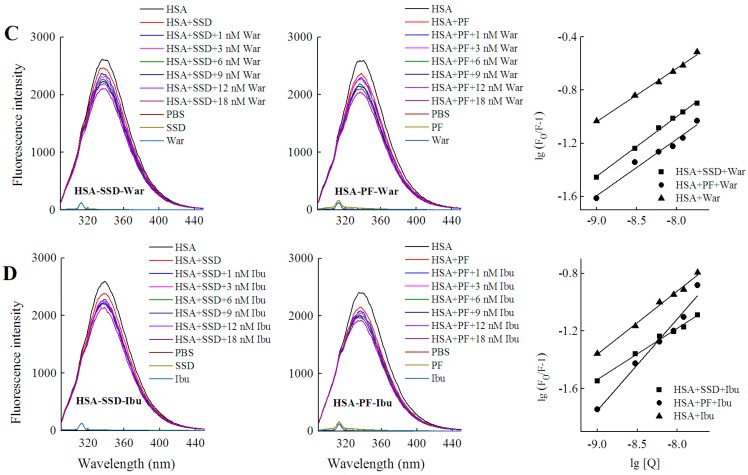
Effect of site marker probes on HSA-SSD and HSA-PF binding at 20 °C. (**A**,**B**) stand for the competition of War/Ibu with 1–18 nM SSD/PF at C_HSA_ = 1 μM, C_War_ = 10 nM, C_Ibu_ = 184 nM; (**C**,**D**) are for the competition of SSD/PF with 1-18 nM War/Ibu at C_HSA_ = 1 μM, C_SSD_ = 3 μM, C_PF_ = 3 μM; [Q], C_SSD_ or C_PF_ or C_SSD_ or C_SSD_ = 0, 1, 3, 6, 9, 12 and 18 nM, respectively. War, Warfarin; Ibu, Ibuprofen; SSD, saikosaponin D; PF, paeoniflorin.

HSA has three homologous domains (I, II, and III): I (residues 1–195), II (196–383), and III (384–585). Each domain contains two subdomains, A and B, which consist of a hydrophobic cavity. Subdomain IIA and IIIA are also called Sudlow I (site I) and Sudlow II (site II), respectively. Site I gives priority to binding with heterocyclic ligands, such as warfarin. Site II more actively binds indole and aromatic compounds, such as ibuprofen [36]. Warfarin and ibuprofen are routinely used to probe sites I and II, respectively [37]. As shown in Figure 7 and Table 6, quenching of HSA fluorescence intensity by about 20% and a blue shift were also observed in all groups. When the concentration of the probe was fixed, the shift induced by HSA-warfarin-SSD or HSA-ibuprofen-SSD mixtures was consistent with the HSA-SSD control, while the shift induced by HSA-warfarin-PF or HSA-ibuprofen-PF mixtures was consistent with the HSA-PF control, which suggests that the probe likely has little effect on the conformational changes of binary binding. When the concentration of SSD/PF is fixed, the shift displayed by the HSA-SSD/PF-probe mixture was consistent with HSA-probe control, suggesting that the probe also exerted little effect on the conformational changes of binary binding.

Among the binary reactions, the *K_a_* for HSA-PF binding was the highest. The *K_a_* for the binding of HSA to warfarin or ibuprofen was 2.54 × 10^2^ and 4.10 × 10^2^, respectively, which is significantly lower than that of the previous report, for micromolar levels of warfarin or ibuprofen, that the *K_a_* values for binding to albumin were 3.4 × 10^5^ and 3.5 × 10^6^, respectively [38]. The difference may be associated with employed methods, drug concentration, reaction system, and experimental conditions. At a fixed concentration of the probe, the *K_a_* of HSA-warfarin/ibuprofen-SSD was significantly lower than that of the HSA-SSD/PF mixtures. At a fixed concentration of SSD/PF, the *K_a_* of the HSA-SSD/PF-probe was higher than that of the HSA-SSD/PF mixtures. It is particularly noteworthy that the *K_a_* of the HSA-PF-probe mixture significantly increased up to the order of 10^5^. These results indicate that SSD/PF, especially PF, promotes the affinity among the ternary complex. Recent observations indicate that warfarin has one high-affinity binding site and several low-affinity binding sites in serum albumin although numerous studies have focused on the high-affinity interaction [39]. Similarly, the decreased binding of ibuprofen is mainly attributed to the competition of low-affinity binding sites of HSA in the presence of acetonitrile [32].This implies that competitive binding most likely occurs at the low-affinity binding sites of HSA. In brief, our site-competitive experiments reveals that both SSD and PF are able to share a common binding site in HSA with warfarin and ibuprofen, namely sites I and II. PF at 10 μM has no binding to site II and ibuprofen at 0–25 μM [24]. The results from high-performance affinity chromatography demonstrate that paeonol interacts with Sudlow site II on HSA [29]. This difference is probably related to experimental conditions, reaction system, or concentrations of PF.

Warfarin (an anticoagulant) is widely used in cardiovascular, cerebrovascular, and other thrombotic accidents [40]. Ibuprofen (a classical anti-inflammatory drug) and its derivatives are also among the most commonly prescribed drugs in the world [41]. As a well-known couplet medication, the combination of Chai Hu with Shao Yao is often prescribed for digestive, gynecological, cardiovascular, and cerebrovascular disease [4,42,43]. Inevitably, the combination of SSD/Chai Hu or PF/Shao Yao often occurs clinically with warfarin- or ibuprofen-class drugs. As an excellent carrier, HSA plays an important role in the release of free drugs. Free drugs are responsible for pharmacological activities [26]. As shown in Table 5, both the *K_a_* of the ternary mixture for warfarin and ibuprofen can be much lower than that of HSA-SSD or -PF binding, suggesting that the concentrations of free SSD or PF will be greatly elevated when the two medicinal herbs are simultaneously combined with warfarin or ibuprofen. In other words, the effect of SSD/Chai Hu or PF/ Shao Yao is likely strengthened when these drugs are co-administered at the same time.

#### 2.4.3. Binding Forces between HSA and SSD/PF

In a thermodynamic process, enthalpy change (Δ*H*) and entropy change (Δ*S*) act as the mainstay to judge their binding force. The major forces of interaction between HSA and drugs include hydrogen bonds, van der Waals forces, electrostatic forces and hydrophobic interactions. The relations of thermodynamic parameters and binding forces are as follows: Δ*H* < 0 and Δ*S* < 0 illustrates the formation of hydrogen bonding or van der Waals forces, Δ*H* < 0 and Δ*S* > 0 indicates an electrostatic force, and Δ*H* > 0 and Δ*S* > 0 indicates hydrophobic interaction [44,45]. When ΔH has no obvious change with temperature, the related parameters can be calculated by the following equations [30]:

(5) $$\ln K_{\alpha }=-\Delta H/RT+\Delta S/R$$

(6) $$\Delta G=-RT\ln K_{a}$$

(7) ΔG=ΔH−TΔS

Here, *K_a_* is the binding constant calculated by Equation (4), and *R* is the gas constant, equaling 8.314 J·K^−1^·mol^−1^ [46,47]. As shown in Table 7, Δ*H*, Δ*S* and free energy change (Δ*G*) are all negative. The negative Δ*G* indicates that the binding is spontaneous. The present negative values of both Δ*H* and Δ*S* reveal that hydrogen bonds and van der Waals interactions play a major role in the thermodynamic process. A previous report by Yu et al. that the main force between paeonol and HSA is hydrogen bonding or van der Waals forces may support our current results (Table 5). Unlike to our partial findings, Δ*H* < 0 and Δ*S* > 0 occurs in the interaction of HSA at high concentrations with PF (Table 5). According to its thermodynamic and docking data, the study explains that HSA-PF binding is dominated by hydrophobic forces as well as hydrogen bonding. This is completely identical to the results of Wen et al. [34], who reported that hydrophobic forces (Δ*H* > 0 and Δ*S* > 0) are responsible for HSA-PF associations (Table 5). Clearly, this needs to be further investigated in order to understand the reason for this discrepancy. Similar to SSD, the value of Δ*G*, Δ*H*, Δ*S* for SSC-HSA binding are all negative, meaning that SSC-HSA association is mainly driven by hydrogen bonding and van der Waal forces (Table 5).

### 2.5. Energy Transfer Resulting from HSA-SSD and HSA-PF Interaction

In this study, the process conformed to three conditions of Förster dipole-dipole non-radiative energy transfer theory. First, HSA served as an endogenous fluorescence donor. Second, there was overlap, to some extent, between the fluorescence emission spectra of the donor and the UV-vis absorbance spectra of the acceptor. Last, HSA and drug were close enough that the largest distance between them was less than 7 nm. Accordingly, HSA-SSD/PF binding involved non-radiative energy transfer, leading to fluorescence quenching. The energy transfer (*E*) is described by Equation (8) [25]:

(8) $$E=1-\frac{F}{F0}=\frac{R_{0}^{6}}{R_{0}^{6}+r^{6}}$$

Here, *F*_0_ and *F* represent the fluorescence intensity of HSA with and without SSD/PF, respectively. The *r* is the distance between donor (HSA) and acceptor (SSD/PF), and *R*_0_ is the critical distance when the efficiency of energy transfer is 50% [25]. *R*_0_ is given by Equation (9) [46]:

(9) $$R_{0}^{6}=8.79\times 10^{-25}\;K^{2}N^{-4}\;\phi J$$

There, *K*^2^ is the dipole spatial orientation factor, *K*^2^ = 2/3. *N* is the refractive index of the medium, equal to the average value of water and organic matter. *φ* represents the fluorescence quantum yield of the donor. In the present work, *K*^2^ = 2/3, and *N* = 1.336. *φ* = 0.118 [47]. *J* is the overlap integral between the fluorescence emission spectrum of the donor and the absorption spectrum of the acceptor, as calculated by Equation (10) [48]:

(10) $$J=\frac{\sum F\left(\lambda \right)\varepsilon \left(\lambda \right)\lambda^{4}\Delta \lambda }{\sum F\left(\lambda \right)\Delta \lambda }$$

**Figure 8:**
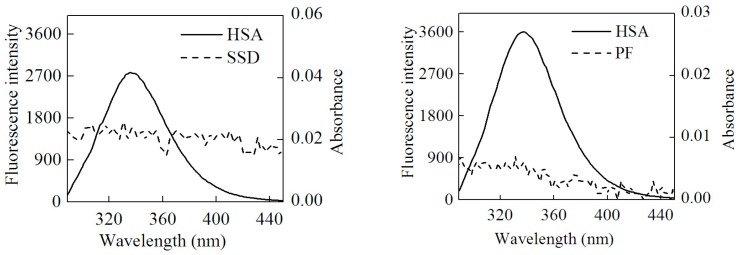
Overlap of the UV absorption spectra of SSD/PF and the fluorescence emission spectra of HSA.

Here, *F*(*λ*) is the fluorescence intensity of the donor and the molar absorption of the acceptor at wavelength *λ*, respectively. *J* can be obtained by integrating the spectra in Figure 8. Then, *R*_0_, *E* and *r* can be worked out. Table 7 shows that all *r* values were between 2 and 7 nm, and 0.5*R*_0_ < *r* < 1.5*R*_0_, which is in accordance with non-radioactive energy transfer theory [25]. This may explain why the fluorescence of HSA was accompanied by quenching with the efficient energy transfer in the interaction of HSA with SSD/PF. In line with the present finding, the previous study also suggests the occurrence of fluorescence energy transfer [24].

### 2.6. Molecular Docking Studies

**Figure 9:**
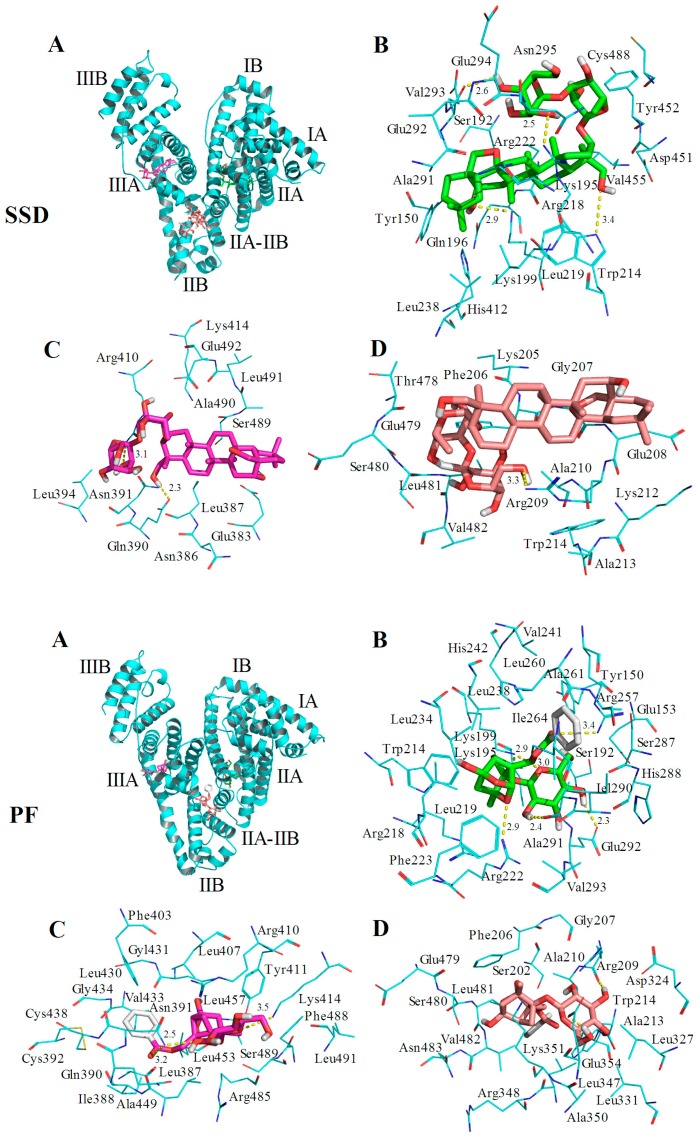
Optimal binding conformation of the SSD/PF-HSA complex (**A**). Amino acid residues of site I (**B**), site II (**C**) or the IIA-IIB site (**D**) surrounding SSD within 4Å. The cyan structure represents HSA, the green, pink or brown stands for SSD/PF, and the yellow dotted line stands for hydrogen bonding.

HSA has a single tryptophan (Trp214) located in site I. As shown in Figure 2, the quenching of PF-mediated HSA is lower than SSD-mediated quenching, suggesting that the HSA-PF binding site is not located at Trp214. To investigate this and the theoretical binding data, molecular docking was employed. Based on the information provided by the PBD databank, SSD was docked to HSA. Seven hydrogen bonds were formed between SSD and site I, site II, and the IIA-IIB binding site of HSA (Figure 9 and Table 8). 

Among these, one hydrogen bond was observed between the Trp214 residue in binding site I and SSD, the pocket of which was surrounded by residues Tyr150 and Tyr452. Additionally, eleven hydrogen bonds were formed between PF and HSA sites I, II, and IIA-IIB. Hydrogen bonds were not found between PF and tyrosine/tryptophan residues although the pocket was surrounded by Trp214 and Tyr150 in site I, and Tyr411, and Trp214 in site IIA-IIB. This is perhaps a reason why the effect of SSD on the quenching of HSA is slightly higher than that of PF. In short, SSD directly binds Trp214, whereas PF does not directly bind to Trp or Tyr. Consistent with the thermodynamic observations, the docking studies demonstrate that hydrogen bonds play a major role in the binding process and the stability of HSA-SSD or -PF complexes. The experimental and docking results both disclose that SSD/PF is mainly docked to sites I and II.

Moreover, SSD/PF is also docked to the interface between sub-domain IIA-IIB, which suggests that the potential binding site may have little effect on the allosteric nature of HAS-SSD/PF binding. Out of these sites, site I is likely to be the most preferred, based on the findings of docking and site-competitive binding. In the previous model, PF is also docked to site I [21]. There is no evidence in the current study yet to show the probability for the competitive binding of SSD/PF to HSA with warfarin or ibuprofen at site IIA-IIB, although the model study points out the potential allosteric nature of SSD/PF. In a word, these results indicate that docking analysis supports a structural basis for the HSA-SSD or -PF interaction.

## 3. Materials and Methods

### 3.1. Materials

HSA (96.0% purity by electrophoresis), tyrosine and tryptophan (both 98.0% purity by TLC) were purchased from Sigma (St. Louis, MO, USA). Commercial prepared SSD and PF (98.0% purity by HPLC) was acquired from Biopurify Phytochemical Ltd. (Chengdu, Sichuan, China). Ibuprofen and warfarin (both 99.7% purity) were purchased from the National Institute for Drug Control (Beijing, China). All other reagents were analytically pure grade.

### 3.2. Absorbance Measurements

The UV-visible absorption spectrum was recorded at room temperature on a NanoDrop 2000c spectrophotometer (Thermo, Waltham, MA, USA) equipped with quartz cells.

### 3.3. Fluorescence Spectra

All fluorescence measurements were recorded on an F-7000 spectrofluorometer (Hitachi, Tokyo, Japan). For conventional fluorescence measurements, the width of the excitation and emission slit were fixed at 2.5 nm and scan speed at 1200 nm per minute and an excitation wavelength at 280 nm [25]. HSA at 1 μM was dissolved in 0.01 M phosphate-buffered saline (PBS), pH 7.4, added to the quartz cell, and then titrated with a 3 μM solution of SSD/PF to reach a concentration of 0 to 18 nM at 20 °C (=293 °K) and 30 °C (=303 °K). For synchronous fluorescence measurements, the excitation and emission slit were fixed at 2.5 nm and the scan speed set at 240 nm per minute. Data were collected with a Δλ = 15 nm for tyrosine and Δλ = 60 nm for tryptophan residues. In the ternary interaction experiments, following 3 μM PF addition to 1 μM HSA, the solution was titrated with 3 μM SSD. The converse experiment was also performed where PF was used to titrate the solution following 3 μM SSD addition to a 1 μM HSA solution. In the site-competitive study, 10 nM warfarin or 184 nM ibuprofen was dissolved in 3 mL of HSA at 1 μM. Then, a SSD/PF solution was successively titrated as indicated in the text. The converse site-competitive experiment was also performed [24,49]. In brief, the concentration of HSA and SSD/PF was fixed at 1 μM and 3 μM, respectively, followed by the titration of 3 μM warfarin or 3 μM ibuprofen. For the direct interaction experiments, 3.0 mL solutions of tyrosine or tryptophan at 10 μM were titrated by successive additions of 3 μM SSD/PF solution.

### 3.4. Correction of the Internal Filter

In order to avoid an inner filter effect, all fluorescence data was corrected using the following formula [25]:

(11) $$F_{\mathrm{cor}}=F_{\mathrm{obs}}\times 10^{\left(A_{\mathrm{ex}}+A_{\mathrm{em}}\right)/2}$$

Here, *F_cor_* and *F_obs_* are the fluorescence intensities of the solution after and before the correction. *A_ex_* and *A_em_* are the absorbances of the solution at the excitation and emission wavelengths, respectively.

### 3.5. CD Spectra

CD spectra of HSA in the absence and presence of SSD/PF were measured by a MOS 450 automatic spectropolarimeter (BioLogic Science Instruments, Claix, France). HSA at a concentration of 1 μM was used. The ratio of HSA, SSD and PF was 1:1:1. A scan rate was set as 30 nm per minute with a response time of 4 s.

### 3.6. Molecular Docking

The three-dimensional coordinates of HSA (PDB ID: 2BXA for SSD; 2BXE for PF) were downloaded from the Protein Data Bank (http://www.rcsb.org/pdb) [50]. The docking input files were produced by The Auto Dock Tools software version 1.5.6 (http://mgltools.scripps.edu). Ligand structures were prepared for docking by merging non-polar hydrogen atoms and defining rotatable bonds. The value of exhaustiveness was set to 20 to promote docking accuracy. For Vina docking, the default parameters were used as described in the Autodock Vina manual unless otherwise specified. The top ranked conformation, as judged by the Vina docking score, was subject to visual analysis by using PyMOL 1.7 software (http://www.pymol.org).

## 4. Conclusions

Both SSD and PF can dose-dependently quench the intrinsic fluorescence of HSA by a static mechanism. Co-binding of SSD with PF also produced a quenching effect on HSA, accompanied by a blue shift of the spectra. The Stern-Volmer constant for HSA-SSD or HSA-PF binding with a low affinity is inversely correlated with temperature. The negative value of ∆*G*, ∆*H*, and ∆*S* indicate that the binding process occurs spontaneously, and is mainly driven by hydrogen bonds and Van der Waals forces. There is a non-radiation energy transfer based on the Förster theory. Experimental site-competitive studies suggest that SSD or PF can bind to site I and II of HSA. These results obtained from synchronous fluorescence and CD spectroscopy demonstrate that SSD or PF are able to change the conformation of HSA when they interact. Docking studies support the experimental and calculated data for conformational change, binding site and binding force induced by SSD or PF. In addition, SSD or PF can also be docked to the interface between site IIA-IIB.

## Figures and Tables

**Figure 1:**
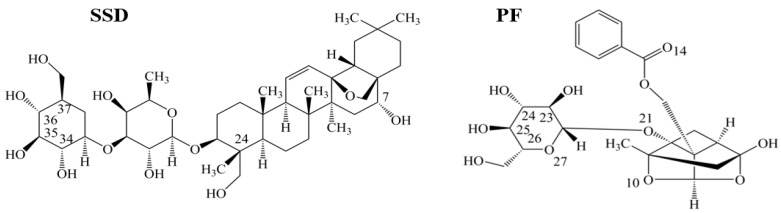
Chemical structures of saikosaponin D (SSD, C_42_H_68_O_13_, MW780.99) and paeoniflorin (PF, C_23_H_28_O_11_, MW480.46), numbered with ChemDraw software.

**Table 1:** Alternation of HSA fluorescence spectra.

Type	Binding	Detection	Shift and Its Range (nm)	Quenching (%)
Binary	HSA-SSD	λex = 280 nm	Left shift 5, 338–333	26.55
		Δλ = 15 nm	No shift	7.43
		Δλ = 60 nm	Left shift 2, 279–277	24.34
	HSA-PF	λex = 280 nm	Left shift 3, 337–334	19.42
		Δλ = 15 nm	No shift	8.29
		Δλ = 60 nm	Left shift 2, 279–277	17.66
Terniary	HSA-PF-SSD	λex = 280 nm	Left shift 13, 339–326	28.63
	HSA-SSD-PF	λex = 280 nm	Left shift 3, 336–333	18.19
Tyrosine	Tyr-SSD	Δλ = 15 nm	No shift	2.82
	Tyr-PF	Δλ = 15 nm	No shift	4.31
Tryptophan	Trp-SSD	Δλ = 60 nm	No shift	12.82
	Trp-PF	Δλ = 60 nm	No shift	10.38

**Table 2:** Percentages of secondary structure before and after binding between HSA and SSD/PF.

Binding	Ratio	α-Helix	β-Sheet	β-Turn	Random Coil
HSA		52.7	7.3	10.9	29.3
HSA-SSD	1:1	46.3	9.0	16.9	27.8
HSA-PF	1:1	55.6	6.5	9.1	25.2
HSA-PF-SSD	1:1:1	66.4	5.0	8.6	20.1

**Table 3:** Stern-Volmer quenching reactive constants.

Binding	Fluorescence	T (°C)	Detection	*K*q (L·mol^−1^·s^−1^)	R
HSA-SSD	Conventional	20	λex = 280 nm	1.6 × 10^15^	0.998
		30	λex = 280 nm	1.1 × 10^15^	0.996
	Synchronous	20	Δλ = 15 nm	4.3 × 10^14^	0.991
		20	Δλ = 60 nm	1.3 × 10^15^	0.994
		30	Δλ = 15 nm	3.5 × 10^14^	0.993
		30	Δλ = 60 nm	1.1 × 10^15^	0.991
HSA-PF	Conventional	20	λex = 280 nm	1.3 × 10^15^	0.995
		30	λex = 280 nm	1.1 × 10^15^	0.993
	Synchronous	20	Δλ = 15 nm	7.5 × 10^13^	0.992
		20	Δλ = 60 nm	7.0 × 10^13^	0.992
		30	Δλ = 15 nm	3.2 × 10^14^	0.994
		30	Δλ = 60 nm	5.8 × 10^14^	0.995
HSA-PF-SSD	Conventional	20	λex = 280 nm	8.5 × 10^14^	0.992
		30	λex = 280 nm	7.3 × 10^14^	0.994
HSA-SSD-PF	Conventional	20	λex = 280 nm	5.9 × 10^14^	0.992
		30	λex = 280 nm	6.8 × 10^14^	0.993

R, linear correlation coefficient.

**Table 4:** SSD- and PF-induced binding reaction parameters of HSA.

Binding	T (°C)	*K_a_* (mol·L^−1^) ± SD	*n*	R
HSA-SSD	20	6.54 × 10^3^ ± 0.146	0.525	0.998
	30	8.28 × 10^2^ ± 0.015	0.464	0.998
HSA-PF-SSD	20	2.46 × 10^3^ ± 0.138	0.497	0.996
	30	0.95 × 10^2^ ± 0.121	0.230	0.996
HSA-PF	20	7.39 × 10^4^ ± 0.174	0.736	0.998
	30	4.43 × 10^3^ ± 0.019	0.482	0.995
HSA-SSD-PF	20	9.58 × 10^2^ ± 0.024	0.324	0.992
	30	3.24 × 10^2^ ± 0.031	0.263	0.991

*K_a_*, binding constant; *n*, the number of binding sites; R, linear correlation coefficient; SD, standard deviation.

**Table 5:** SSD/PF-related compound Ka and thermodynamic parameters.

Drug	°C	*K_a_*	*n*	Δ*G*	Δ*S*	Δ*H*	Reaction System	Reference
PF	25	3.56 × 10^4^	0.62	−21.0	103.5	4.1	10–70 μM PF, 10 μM BSA, pH7.4 Tris-HCL	[34]
	37	3.78 × 10^4^	0.61	−22.0				
PF	15	1.91 × 10^3^	0.91	−18.1	28.2	−10.0	2.5–12.5 μM PF, 10 μM BHSA, pH7.4 Tris-HCL	[24]
	37	1.42 × 10^3^	0.89	−18.7				
Paeonol	10	1.58 × 10^4^	1.01	−22.7	88.7	2.4	10–70 μM paenol, 1 μM BHSA, pH7.4 Tris-HCL	[35]
	40	9.68 × 10^2^	0.84	−17.9	64.9			
Paeonol	4	1.33 × 10^4^	-	−22.0	−6.3	−23.8	pH7.4 PBS	[28]
	37	3.55 × 10^3^	-	−21.8				
SSC	26	3.72 × 10^3^	0.79	−20.34	−90.3	−47.3	1–15 μM PF, 2 μM BHSA, pH7.4 PBS	[26]

*K_a_*, mol·L^−1^; Δ*G*,kJ·mol^−1^; Δ*S*, J·mol^−1^·K^−1^; Δ*H*, kJ·mol^−1^; PF, paeoniflorin; SSC, saikosaponin C.

**Table 6:** Binding reaction parameters in site-competitive experiments.

Fixed	Binding	Shift and Range (nm)	Quenching (%)	*K_a_* (mol·L^−1^) ± SD	*n*	R
Probe	HSA-SSD	Left shift 5, 338–333	26.55	6.54 × 10^3^ ± 0.146	0.525	0.998
	HSA-War-SSD	Left 5, 337–332	29.93	3.18 ± 0.016	0.224	0.995
	HSA-Ibu-SSD	Left 5, 338–333	26.12	8.42 ± 0.025	0.177	0.992
	HSA-PF	Left shift 3, 337–334	19.42	7.39 × 10^4^ ± 0.174	0.736	0.998
	HSA-War-PF	Left 3, 338–335	18.57	3.45 ± 0.036	0.277	0.997
	HSA-Ibu-PF	Left 2, 336–334	16.09	7.86 ± 0.039	0.382	0.994
SSD/PF	HSA-War	Left 3, 338–335	19.07	2.54 × 10^2^ ± 0.899	0.389	0.996
	HSA-PF-War	Left 4, 340–336	14.78	4.11 × 10^5^ ± 3.420	0.716	0.997
	HSA-SSD-War	Left 4, 339–335	17.00	9.72 × 10^2^ ± 0.448	0.469	0.996
	HSA-Ibu	Left 3, 338–335	17.07	4.10 × 10^2^ ± 0.169	0.448	0.992
	HSA-SSD-Ibu	Left 1, 335–334	17.08	7.21 × 10^3^ ± 0.368	0.520	0.994
	HSA-PF-Ibu	Left 3, 338–335	19.36	4.2 × 10^5^ ± 0.034	0.638	0.992

This study was carried out at 20 °C. λex = 280 nm; *n*, number of binding sites; SD, standard deviation.

**Table 7:** Thermodynamic and energy transfer parameters.

Binding	T (°C)	Δ*G* (kJ·mol^−1^)	Δ*S* (J·mol^−1^·K^−1^)	Δ*H* (kJ·mol^−1^)	*J* (cm^3^·L·mol^−1^)	*R*_0_ (nm)	*E* (J)	*r* (nm)
HSA-SSD	20	−19.33	−314.92	−111.60	1.03 × 10^−14^	2.50	0.24	3.00
	23	−17.44			1.03 × 10^−14^	2.47	0.21	3.07
	27	−16.45			1.04 × 10^−1^^4^	2.06	0.13	2.83
	30	−16.18			1.04 × 10^−14^	2.47	0.27	2.92
HSA-PF	20	−27.64	−1201.16	−379.58	1.88 × 10^−15^	1.86	0.05	2.27
	23	−22.19			1.91 × 10^−15^	2.02	0.08	3.02
	27	−21.29			1.91 × 10^−15^	2.05	0.12	2.85
	30	−15.63			1.92 × 10^−15^	1.86	0.23	2.27

**Table 8:** Atoms and amino acid residues involved in hydrogen bonds formed between HSA and SSD/PF.

	Site	Amino Acid Residue	Atoms *	Bond Length	Trp or Tyr around the Pocket
SSD	Site I	Lys199	CH_2_OH (7)	2.9 Å	Tyr150 Tyr452
		Trp214	CH_2_CH_2_OH (24)	3.4 Å	
		Arg218	CH_2_OH (35)	2.5 Å	
		Glu292	CH_2_OH (36)	2.6 Å	
	Site II	Asn391	CH_2_CH_2_OH (24)	2.3 Å	Trp214
		Arg410	CH_2_OH (34)	3.1 Å	
	IIA-IIB	Arg209	CH_2_CH_2_OH (37)	3.3 Å	
PF	Site I	Lys199	O (21)	2.9 Å	Tyr150 Trp214
		Lys199	O (27)	3.0 Å	
		Arg257	O (14)	3.4 Å	
		Arg218	CH_2_OH (25)	2.3 Å	
		Ala291	CH_2_OH (23)	2.4 Å	
		Glu292	O (10)	2.9 Å	
	Site II	Asn391	O (14)	3.2 Å	Trp214
		Asn391	CH_2_OH (23)	2.5 Å	
		Lys414	O (27)	3.5 Å	
	IIA-IIB	Arg209	CH_2_OH (24)	2.2 Å	Tyr411
		Glu354	CH_2_CH_2_OH (26)	2.0 Å	

* see Figure 1.
